# Structural Implications of Missense Point Mutations in Shwachman–Bodian–Diamond Syndrome Protein (SBDS): A Combined SAXS/MD Investigation

**DOI:** 10.1021/acsomega.5c04764

**Published:** 2025-08-01

**Authors:** Giovanni Mattiotti, Vittoria Nanna, Marco Giulini, Domenico Alberga, Giuseppe Felice Mangiatordi, Nuria Sánchez-Puig, Michele Saviano, Luca Tubiana, Raffaello Potestio, Gianluca Lattanzi, Dritan Siliqi

**Affiliations:** † Physics Department, 19034 University of Trento, Via Sommarive 14, I-38123 Trento, Italy; ‡ INFN-TIFPA, Trento Institute for Fundamental Physics and Applications, I-38123 Trento, Italy; § 296400 CNR - Istituto di Cristallografia, Via Amendola 122/o, I-70126 Bari, Italy; ∥ Instituto de Química, Universidad Nacional Autónoma de México, Circuito Exterior s/n, Ciudad Universitaria, México City 04510, México; ⊥ CNR - Istituto di Cristallografia, URT Caserta, Via Vivaldi 43, I-81100 Caserta, Italy

## Abstract

Shwachman–Diamond syndrome (SDS) is a rare autosomal recessive disorder characterized by pleiotropic phenotypes, including pancreatic insufficiency, skeletal abnormalities, and bone marrow dysfunction. Notably, patients with SDS exhibit an increased risk of developing myelodysplastic syndrome and leukemia. In this study, we employed a combination of comparative molecular dynamics (MD) simulations and small-angle X-ray scattering (SAXS)-based analysis to investigate the Shwachman–Bodian–Diamond syndrome protein (SBDS). Specifically, we explored the molecular basis of the syndrome by examining the conformational dynamics of a set of missense mutants of SBDS in comparison to those of the wild-type (WT) protein. Our observations suggest that different mutations may impact (i) the interaction of SBDS with the ribosome, (ii) the binding of SBDS to Elongation Factor-Like 1 (EFL1), and (iii) the SBDS rearrangements coupled to EFL1 binding. Extensive MD simulations, with a total simulation time of 17 μs, revealed variations in the interdomain flexibility of SBDS, which are consistent with previously published affinity data and the new SAXS experimental data presented here. We propose a structural rationale behind the previously reported weak interaction of mutants I167T, R175W, and I212T with EFL1. Additionally, SAXS data indicate that R19Q, I167T, and R175W mutants exhibit altered relative abundances of SBDS conformational states in solution, further supporting our computational results. Overall, our integrated computational and experimental approach provides a comprehensive understanding of how specific mutations in SBDS alter its structural dynamics and binding interactions. These insights enhance our broader understanding of SBDS function and its role in ribosome biogenesis.

## Graphical abstract

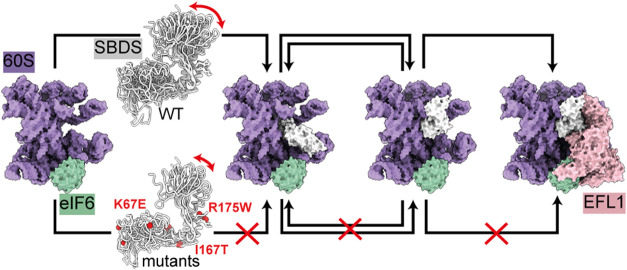

## 1 Introduction

Shwachman–Diamond syndrome (SDS) is a rare autosomal recessive disorder characterized by pleiotropic phenotypes, including pancreatic insufficiency, skeletal abnormalities, and bone marrow dysfunction.^1,2^ In addition, patients with SDS also exhibit an increased risk of myelodysplastic syndrome and leukemia.^1^ In over 90% of cases, SDS is caused by biallelic loss-of-function mutations in the Shwachman–Bodian–Diamond syndrome protein (SBDS).^3^ Most patients display the biallelic pathogenic variants c.183_184TA > CT (K62X) and c.258 + 2T > C (C84 fsX3), which produce truncated versions of SBDS.^3^ Other less common mutations have also been reported, including nonsense mutations, missense mutations, small deletions, indel conversions, and splice-site mutations.^4–7^ Interestingly, less than 10% of SDS patients do not harbor mutations in the *SBDS* gene but instead have variants in other genes encoding proteins such as DNAJC21,^8,9^ SRP54,^10^ and the GTPase Elongation Factor-Like 1 (EFL1).^11–15^ SBDS and EFL1 are essential for the assembly and biogenesis of the 60S ribosomal subunit in the cytoplasm. Together, they release the eukaryotic Initiation Factor 6 (eIF6) from the 60S preribosomal subunit in a GTP-dependent manner (Figure 1A). eIF6 plays a critical role in regulating ribosome assembly by binding to the 60S subunit, where it sterically blocks the B6 intersubunit bridge, preventing the association of the 40S and 60S subunits to form a functional 80S ribosome.^16,17^ The release of eIF6 not only facilitates its recycling to the nucleus but also ensures proper processing of pre-rRNA precursors.^18^ In current models of this process, SBDS is a crucial factor: it is recruited to the pre-60S-eIF6 particle and assesses the integrity of the P-site through domain I, while domain III contacts the Sarcin–Ricin Loop (SRL) and the base of the P-stalk. Although the eIF6 factor binds to the pre-60S subunit near the SRL, it does not contact SBDS. Instead, the space between SBDS and eIF6 is occupied by EFL1, which interacts with the GTPase-associated center (GAC). EFL1, in its inactive GTP-bound state, triggers a structural rearrangement of domains II and III of SBDS. Simultaneously, it adopts an active GTP-bound conformation accommodated in the SRL site that competes with eIF6 for an overlapping binding site on the 60S ribosomal subunit, ultimately displacing it. In this context, SBSD functions as a guanine nucleotide exchange factor (GEF) for EFL1, dramatically decreasing its affinity for GDP and predisposing it to adopt the active GTP-bound conformation.^19^

**Figure 1:**
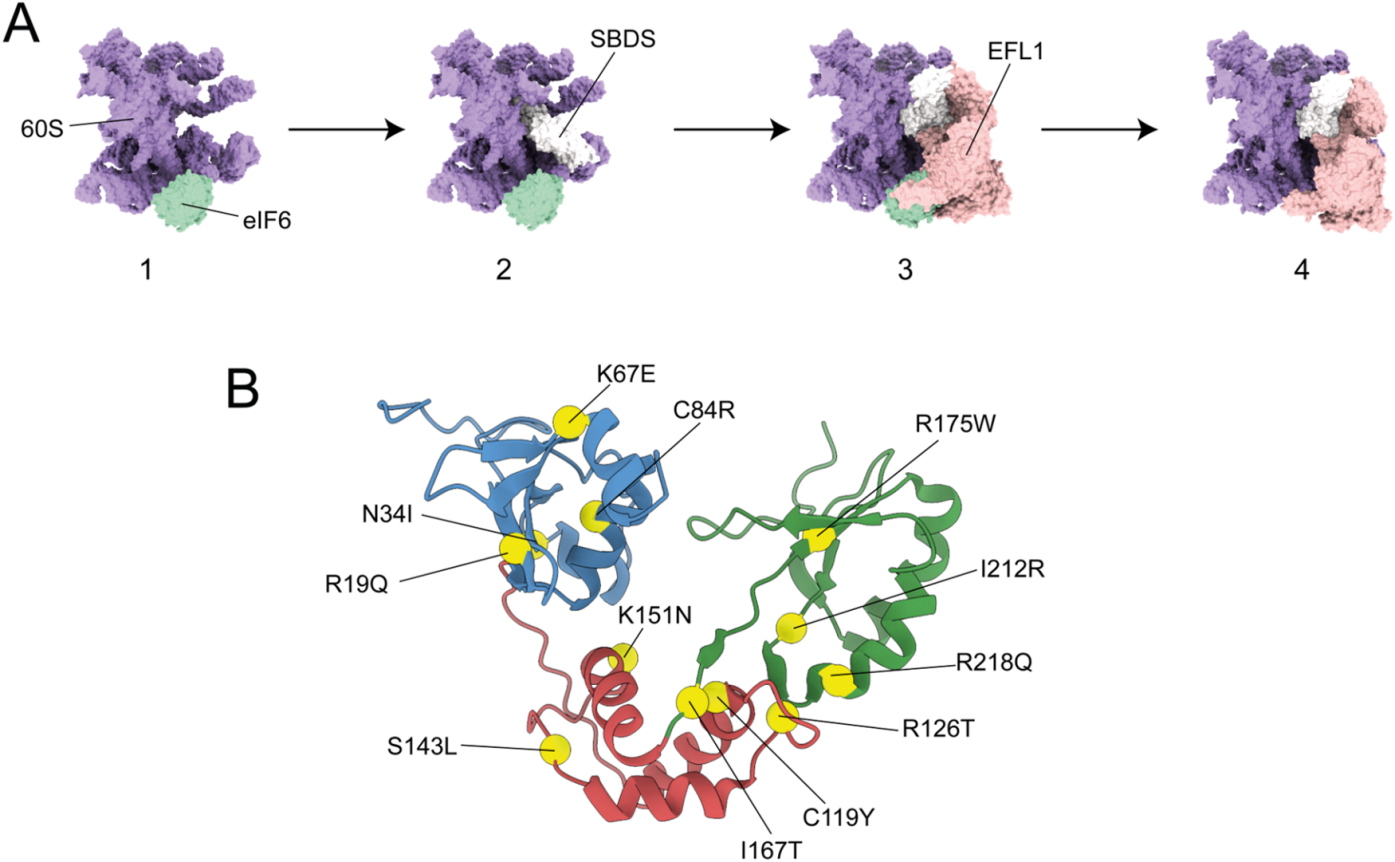
(A) Final steps in the 60S ribosomal subunit maturation:^20^ (1) eIF6 binds to the 60S subunit preventing premature joining to the 40S subunit. (2) SBDS is recruited to the 60S subunit and interacts with it via all its domains. (3) EFL1 interacts directly with SBSD and eIF6. Prior to, or promoted by, the EFL1 binding, SBDS undergoes a structural rearrangement that includes the rotation of SBDS domain III. (4) EFL1 competes with eIF6 for an overlapping binding site, facilitating its displacement from the 60S subunit. The structures shown at each step are based on the cryogenic electron microscopy (cryo-EM) maps published by Weis et al.^20^ (PDB codes: 5AN9, 5ANB, 5ANC). (B) Cartoon representation of the SBDS protein (conformer V from NMR structure ensemble, PDB code: 2KDO^21^). The Cα atoms of the residues whose mutations are studied in this work are shown as yellow spheres. Domain I, blue; Domain II, red; Domain III, green.

It is known that some SBDS missense mutations alter the protein stability and folding, reducing functional protein levels and impairing the activation of EFL1.^22^ However, other variants that do not affect protein folding still lead to clinical manifestations of SDS through largely unknown mechanisms. Several questions remain unanswered: (i) Do these mutations impact SBDS conformation? (ii) If so, how does the altered conformational landscape of mutant SBDS impact ribosomal maturation? (iii) Can we gain molecular insights into the ribosomal maturation process by studying SBDS mutants? A previous coauthored work^23^ provided an initial attempt to address these questions by applying comparative molecular dynamics (MD) to three SBDS missense mutations (R19Q, R126T, and I212T). The data suggested that the SBDS functionality may involve an allosteric mechanism that engages both domain I and domain III of the protein. Encouraged by these findings, we expanded the analysis in this ongoing work by conducting MD simulations on a larger panel of SBDS mutants, including R19Q, N34I, K67E, C84R, C119Y, R126T, S143L, K151N, I167T, R175W, I212T, and R218Q^24,25^ (Figure 1B). The results of our computational investigation, supported by small-angle X-ray scattering (SAXS) data, have provided new insights into how specific mutations alter SBDS function and, consequently, the pathogenesis of SDS.

## 2 Materials and Methods

### 2.1 Molecular Dynamics Simulations

#### 2.1.1 System Setup and Simulation Protocol

The structures of human SBDS available in the Protein Data Bank were obtained by nuclear magnetic resonance (NMR) spectroscopy (PDB code: 2KDO^21^) and cryogenic electron microscopy (cryo-EM) (PDB codes: 5AN9, 5ANB, 5ANC^20^). We selected the NMR structure as the starting point for our studies, as it was resolved in solution, thus offering information about the protein flexibility through an ensemble of conformations.

In a previous report,^25^ we studied the conformational flexibility of the yeast SBDS orthologue in solution and demonstrated that it exists as an ensemble of three primary conformations, which vary in the orientation of domains I and III relative to the central domain II. Similarly, the NMR structure of the human SBDS orthologue revealed a comparable ensemble of conformations, with the most prominent ones featuring domains I and III either in proximity or positioned furthest from domain II. We selected two conformational ensembles (conformer V as open state and conformer II as closed one) to represent the protein’s natural behavior in solution (Supporting Figure 8). These ensembles formed the basis for our MD simulations, providing a realistic and biologically relevant framework for studying the impact of mutations on the SBDS dynamics and function. Using these conformations, we generated 12 missense point mutants,^24–26^ namely, R19Q, N34I, K67E, C84R, C119Y, R126T, S143L, K151N, I167T, R175W, I212T, and R218Q, with the Mutator Plugin (v1.5) of VMD 1.2.3.^27^ This resulted in 26 different systems: 12 mutants and the wild-type form (WT), each in both the open and closed conformations. These systems were used as starting points for MD simulations performed using GROMACS v2018.^28^ The most probable ionization state of the histidine at physiological pH was assigned by GROMACS, and the hydrogen atoms were placed accounting for the local environment. Each structure was solvated in a water box with at least 15 Å separation of the protein atoms from the box edge. K^+^ and Cl^–^ ions were added to neutralize the system and reach a physiological concentration of 0.15 M. We used the Amber ff99SB-ILDN^29^ force field that has been shown to provide reliable conformational stability, which is crucial for studying proteins in defined structural states,^30,31^ and the TIP3P^32^ water model. A cutoff distance of 12 Å was applied for the van der Waals (vdW) and short-range interactions, while long-range electrostatic forces were calculated using the particle-mesh Ewald method.^33^ A 2 fs step was applied for all simulations. Each solvated system underwent energy minimization using the steepest descent algorithm with a maximum tolerance force of 500 kJ/mol nm. Subsequently, the systems were equilibrated at 310 K for 500 ps, restraining the protein’s heavy atoms with a harmonic potential in the minimized configuration, in the NVT ensemble using the modified velocity rescale thermostat.^34^ A further 500 ps equilibration was performed in the NPT ensemble using the Parrinello–Rahman barostat^35^ with a reference pressure of 1 atm. Finally, after removing the restraints on the heavy atoms, we performed 500 ns production runs for each of the 26 systems in the NVT ensemble at 310 K. To complement the experimental SAXS analyses and increase the statistical significance of our results, we simulated two additional independent replicas for the WT, R19Q, I167T, and R175W systems. This brought the cumulative simulation time to 17 μs.

#### 2.1.2 Analyses

Root mean square deviations (RMSD), root mean square fluctuations (RMSF), and solvent-accessible surface area (SASA) were calculated using Gromacs 2018 utilities. The reference structures for the RMSD were the starting frame of the production runs, while those for the RMSF were the average configuration of the production run. For independent replicas (i.e., for the WT, R19Q, I167T, and R175W systems), the RMSF was calculated on the concatenated equilibrated trajectory. For the calculation of the RMSD of the three domains of SBDS, we used the boundaries reported by de Oliveira et al. with domain I corresponding to residues 9–95, domain II to residues 107–167, and domain III to residues 173–236.^21^ The RMSD calculations were performed by aligning all of the trajectories on the Cα atoms of the protein.

##### 2.1.2.1 Cosine Content Analysis

Introduced by Hess,^36^ cosine content quantifies the similarity between the dynamics of a system and a random diffusion process. If we consider the principal components (PCs), *p*_1_(*t*), *p*_2_(*t*), ···, as the dynamical variables, the cosine content is defined as

$$cc\left[p_{i};t_{f}\right]≔\frac{2}{t_{f}}{\left\{\int_{0}^{t_{f}}\cos\left[\left(i+1\right)\pi t\right]p_{i}\left(t\right)dt\right\}}^{2}{\left(\int_{0}^{t_{f}}p_{i}^{2}\left(t\right)dt\right)}^{-1}$$

where *t*_f_ is the ending frame of the trajectory and *i* is an index for the *i*-th PC. For the calculation, we used the function *cosine content* implemented in Python library MDAnalysis, where the frequency *k* in Hess’s equation is set to *i* + 1.^49^ We varied this parameter (*t*_f_ = 100 ns, 200 ns, 300 ns, 400 ns, 500 ns) to compute the cosine content for progressively longer trajectory segments, comparing the values for *t*_f_ = 100 ns and *t*_f_ = 200 ns to identify trends in this quantity. As reported by the Hess,^36^ a critical value of the *cosine content* is assumed to be 0.7; higher values indicate that the trajectory is not equilibrated.

##### 2.1.2.2 Free Energy

Given pairs of observable values, such as the dihedral angles (Φ, Ψ) of an amino acid, the free-energy surface (FES) quantifies the relative stability of the states explored by the system as follows

$$F\left[\Phi ,\Psi \right]=-k_{b}T\;\log\left[\rho \left(\Phi ,\Psi \right)\right]+F_{0}$$

where *k*_b_ is the Boltzmann constant, *T* is the temperature of the system, ρ is the histogram of the collective variables (CVs) Φ and Ψ, and *F*_0_ is an arbitrary constant used to set the zero of the free energy. In this work, we used Python package PyEMMA^37^ to perform free-energy calculations.

##### 2.1.2.3 SASA-Based Binding Affinity Estimator

Given a mutation μ in either the open or closed state (γ), the SASA-based binding affinity estimator α_μ,γ_^(*I*)^ is defined as the sum of the solvent-accessible surface area (SASA) along the trajectory with *T* frames, weighted by the residue charge.

$$\alpha_{\mu ,\gamma }^{\left(I\right)}=\sum_{r=1}^{N_{I}}q_{r}\left\langle \mathrm{SAS}A^{\left(\mu ,\gamma \right)}\right\rangle \equiv \sum_{r=1}^{N_{I}}q_{r}\left(\frac{1}{T}\sum_{t=1}^{T}\mathrm{SAS}A^{\left(\mu ,\gamma \right)}\left(t\right)\right)$$

This calculation was restricted to domain I, as it is the only domain directly involved in interactions with rRNA.^21^

### 2.2 Small-Angle X-ray Scattering (SAXS)

#### 2.2.1 Small-Angle X-ray Scattering (SAXS) Experiments

Recombinant human SBDS protein and three selected mutations (R19Q, I167T, R175W) were expressed in *Escherichia coli* C41 and purified as described in refs 25,38 . All constructs encoded a C-terminal FlAsH tag and an N-terminal 6× histidine tag.

Structural characterization of SBDS and its mutants was performed using size-exclusion chromatography coupled with small-angle X-ray scattering (SEC-SAXS). Data were collected at the EMBL P12 beamline, at PETRA III (Hamburg, Germany),^39^ using a Pilatus 6 M detector at a sample–detector distance of 3 m and a wavelength of λ = 0.124 nm. SEC-SAXS^40^ was performed at 20 °C using a Superdex 75 Increase 5/150 GL size-exclusion column at a flow of 0.3 mL/min and 50 mM Tris-HCl pH 8.0, 10% glycerol, 300 mM NaCl, and 5 mM MgCl_2_ as running buffer. Each sample (50 μL) was injected at concentrations of 11 mg/mL (WT), 13.8 mg/mL (R19Q, I167T), and 13 mg/mL (R175W), and 1800 frames were collected, each with an exposure time of 0.5 s per frame. Raw data were normalized to the intensity of the transmitted beam and radially averaged using integrated software available at the beamline.^39^ Chromixs^41^ software was used to select the frames corresponding to the scattering of the sample and the frames corresponding to the appropriate solvent blank. Subtracted and averaged frames provided the final SAXS profile, enabling detailed structural comparisons between the WT and mutant proteins.

#### 2.2.2 SAXS Data and Flexibility Analysis

Initial SAXS data analysis was performed with the PRIMUS software.^42^ Zero-angle scattering intensity *I*(0) and the radius of gyration *R*_g_ were derived from Guinier plots within the range of 0–1/*R*_g_.^43^ The particle distance distribution function P­(r) and the maximum dimension of the proteins *D*_max_ were determined using the Indirect Fourier Transformation method as described in ref 44. Molecular mass estimates were obtained using PRIMUS.^42^ Detailed data collection information and SAXS parameters are presented in Supporting Table 1. Flexibility studies for all SBDS constructs were performed using the online version of the program MultiFoXS (http://modbase.compbio.ucsf.edu/multifoxs/)^45^ adopting the cryo-EM structure (PDB code: 5ANC, Chain J^20^) as the starting model. The program requires as input the atomic structure of the studied protein, a list of flexible residues, and the corresponding experimental SAXS profile. The rigid parts of the proteins were identified using the HingeProt program,^46^ corresponding to the three domains of SBDS with hinges at residues D97 and A170. MultiFOXS generates 10,000 conformations, calculates the corresponding SAXS profiles, and ranks the best models according to a multistate scoring function (χ). Multistate models were selected based on the best fit between experimental and calculated SAXS profiles. All experimental and modeled SAXS data have been deposited and validated in the Small Angle Scattering Biological Data Bank (SASBDB^47^) under the accession codes SASDVC4 (WT), SASDVD4 (R19Q), SASDVE4 (I167T), and SASDVF4 (R175W).

To compute the angular displacement between SBDS domains, in*-house* PyMOL scripts were used. Specifically, the angle between the center of mass of domains I, II and III was measured. The rotational angle and the displacement of domains I and III in the mutant protein, relative to the compact state for each construct (WT and the three mutants), were quantified using the orientation.py script by Thomas Holder (https://pymolwiki.org/index.php/psico). This script was executed after aligning the three conformers on domain II.

## 3 Results and Discussion

### 3.1 MD Simulations Suggest Altered Interdomain Mobility in SBDS Mutants

As an initial step, we computed the time-dependent RMSD values from all generated trajectories. Our aim was twofold: (i) to identify the equilibration time for the studied systems, and (ii) to gain preliminary insights into the conformational effects of the SBDS mutations. RMSD values were computed solely for the Cα atoms. As shown in Figure 2 and Supporting Figure 1, all systems can be considered equilibrated after the first 100 ns.

**Figure 2:**
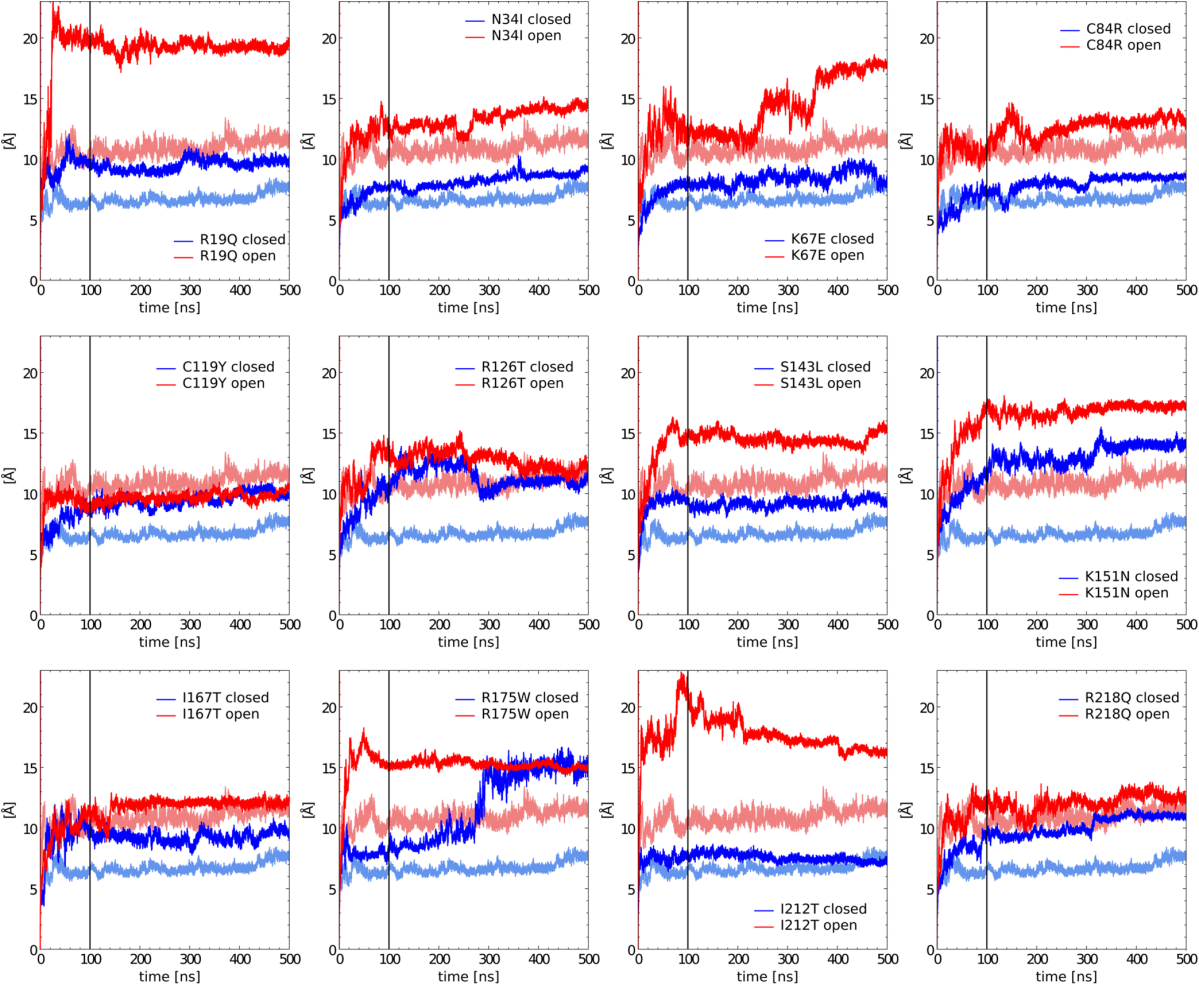
Time dependence of the RMSD values computed on the Cα atoms for all of the investigated SBDS mutants. Blue and red curves represent the trajectories for the closed and open conformations, while light-colored lines show the wild-type data for comparison. The black vertical line separates the equilibration phase from sampling one.

As expected, the systems presenting mutations deviate more significantly from the initial point (as indicated by higher RMSD values) and exhibit fewer stable profiles compared with the two WT conformations. Notable examples include the K67E and I212T open systems. Significant variations were observed in some trajectories, particularly in the R175W closed conformation. In this specific case, the transition may indicate that the protein in this simulation reaches a more stable local minimum after the first 100 ns, around 200 ns. To support the assumption that the R175W closed trajectories are equilibrated after 100 ns, we calculated the cosine content values, a metric used to identify nonrelaxed trajectories using principal component analysis (PCA). The obtained values, reported in the Supporting Figures 2 and 3, fall below the critical range [0.7, 1],^36^ suggesting that the interval between 100 and 500 ns can be deemed equilibrated. This assumption is further supported by the RMSD calculations performed on the Cα atoms for each domain (domains I, II, and III) individually (Supporting Figure 4). To study the effect of the mutations on the relative mobility of the three domains, we computed the RMSF values for each residue (Figure 3).

**Figure 3:**
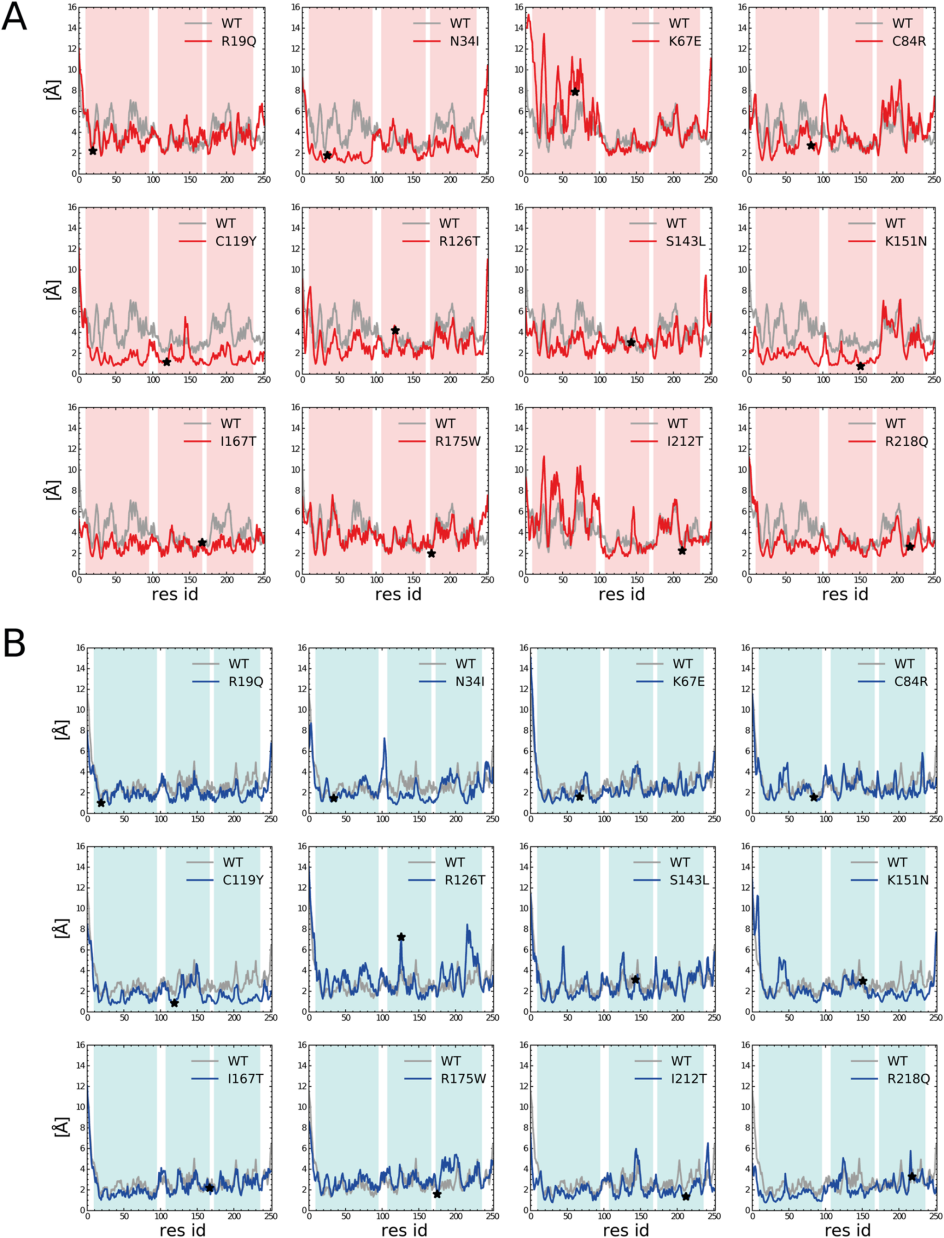
RMSF per residue for the open (A) and closed (B) trajectories for each mutation. The RMSF values for the WT system are shown in gray. Residues corresponding to mutation sites are marked with a black star. The background is color-coded according to the three domains of SBDS: red for the open conformation and light blue for the closed conformation, with the unstructured linker regions are shown in white.

More specifically, we divided the protein into different regions (domain I, hinge I–II, domain II, hinge II–III, and domain III) and studied how much their conformational landscape differs from that observed for the WT form. Our observations are summarized in Table 1.

**Table 1:**
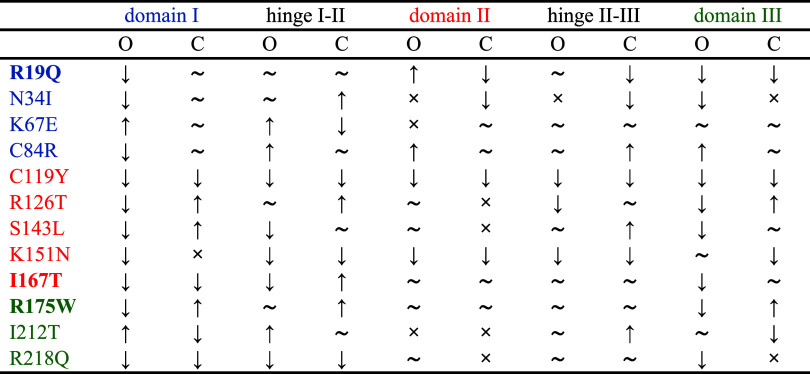
Summary of RMSF Discrepancies between WT and Mutated Trajectories (in Bold Those Studied with SAXS Here), in the Open (O) and Closed (C) States, Analyzed Regionwise along the Protein Sequence^a^

a The symbol ∼ indicates fluctuations comparable to those of the WT, while ×, ↑, and ↓ represent in order: differing fluctuations that are a combination of higher and lower (within the residues of that region), higher, and lower, respectively, considering an average difference of 1 Å as threshold. A color code links the mutated residue to its corresponding domain.

First, in the open conformation, domain I shows consistently reduced mobility in most mutants compared with the WT system, suggesting a general rigidification of this region. The only notable exceptions are K67E and I212T, where increased flexibility is observed, pointing to mutation-specific effects. Interestingly, mutant trajectories in the closed conformation tend to more closely resemble the WT, implying that this state is less affected by mutation-induced changes.

Second, some mutations, such as C119Y and K151N, induce a global reduction in flexibility across all protein domains and both conformations, indicating a rigidifying effect on the protein structure. Among these, C119Y is particularly striking, with uniformly decreased fluctuations throughout, suggesting the formation of a more compact and rigid conformation.

Third, mutation effects on hinge flexibility and interdomain coupling are highly variable and often conformation dependent. While certain mutations (e.g., C84R/open, N34I/closed) increase hinge I–II flexibility, others present reduced or heterogeneous changes. Moreover, mutants such as I167T and R175W display asymmetric fluctuation profiles between open and closed states, suggesting a mutation-induced shift in conformational equilibrium.

These dynamic changes may have functional consequences. The reduced mobility in key structural transitions could hinder the conformational rearrangement required for SBDS to engage productively with EFL1 (Figure 1A, steps 2 to 3). This hypothesis aligns with previously published data on the affinity of SBDS mutants for EFL1. Gijsbers et al.^25^ experimentally demonstrated that the I167T mutation weakens SBDS binding to EFL1, while mutations R175W and I212T disrupt it entirely. While further studies of the SBDS-EFL1 interaction are needed to robustly explain these data, the altered flexibility for the same mutants provides a possible conformational explanation for this experimental evidence. In the presence of the studied mutations, SBDS might not be able to undergo the structural rearrangement required to efficiently interact with EFL1.

### 3.2 Key Mutations Hinder SBDS Binding to RNA

Motivated by the consistency between experimental data and MD simulations, we analyzed the conformational variability sampled during the simulations by calculating the free-energy landscape (FEL) for each mutant in both open and closed conformations. The chosen collective variables (CVs) were the radius of gyration (*R*_g_) and the angle θ, formed by the centers of mass of domains I, II, and III. Figure 4 shows the low-dimensional conformational variability explored by most of the investigated systems, highlighting distinct profiles for each mutation. For instance, the simulations starting from the open conformation of the R19Q mutant reveal a conformational space significantly different from that of the WT. This mutation appears to induce a substantial opening of the structure, suggesting a conformational effect, contrary to prior hypotheses^48^ that attributed its impact to changes in electrostatic potential affecting rRNA binding. This idea is further supported by an analysis of the closed conformation, which also shows an overall more compact conformation for the R19Q mutation. In contrast, no significant conformational changes are observed in the K67E. This is evident across the plots for both the open and closed conformations, supporting the idea that this mutation, which involves the substitution of opposite charged residues, primarily alters electrostatic potential and impacts the interaction with the P-loop, as hypothesized by Weis et al.^20^

**Figure 4:**
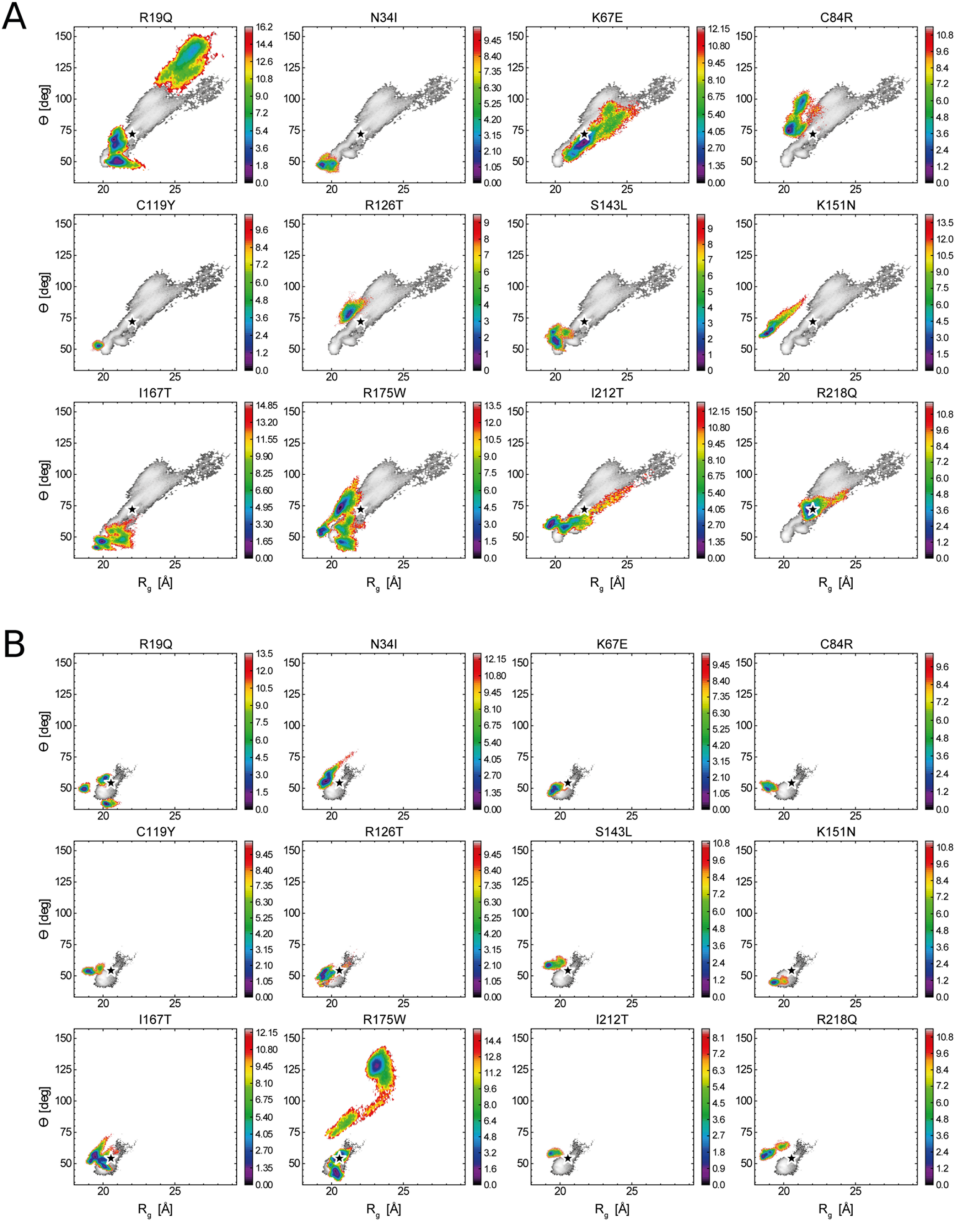
Free-energy surfaces (FES) for various SBDS mutants, built from the simulation histograms *h*[*R*_g_, θ] sampled in the open (A) and closed (B) conformation runs. Each plot shows the FES of the mutant (colored) overlaid with that of the WT (gray). The *x*-axis represents the radius of gyration (*R*_g_), while the *y*-axis represents the interdomain angle (θ). Color maps indicate free-energy values in kcal/mol, calculated at a reference temperature of *T* = 310 K. Black stars denote the positions of the NMR structure used to initiate the MD simulations.

We used the Jensen–Shannon (JS) divergence to quantitatively compare the frequentist probabilities sampled by the mutants and the WT.^49^ Dendrograms were created for the open and closed state runs using JS divergence as a measure of the distance between the domains, which ranges from 0 to 1 (Supporting Figure 5A). The mutual JS distances are reported in a matrix format (Supporting Figure 5B), with values from the open-conformation trajectories shown in the lower-left part and those from the closed ones in the upper-right part. This analysis indicates that the R19Q simulation in the open state and the R175W simulation in the closed state display the most distinct frequentist probability distribution in the (*R*_g_, θ) space.

To test whether specific mutations affect rRNA binding, we computed the solvent-accessible surface area (SASA) of positively and negatively charged residues in domain I for all 26 simulations. The aim was to identify whether specific mutations altered the solvent exposure of charged residues, potentially leading to a higher buriedness of positive residues and greater exposure of negative ones. Such a scenario could explain a decrease in the binding affinity of SBDS for rRNA due to changes in charge-mediated interactions.

We computed the average SASA for each residue in domain I throughout the simulations, as this domain is responsible for rRNA interaction.^21^ We then determined the difference in SASA for each charged residue between mutant and WT runs. These differences and their standard deviations are reported in Supporting Figures 6 and 7. To estimate the total SASA of charged residues in domain I, weighted by their charge, we defined a new metric called the SASA-based binding affinity estimator (α). In this way, positively charged residues favor the interaction with the negatively charged phosphate groups of the rRNA backbone, while negatively charged residues disfavor the interaction due to electrostatic repulsion. The α values for each mutation are listed in Figure 5.

**Figure 5:**
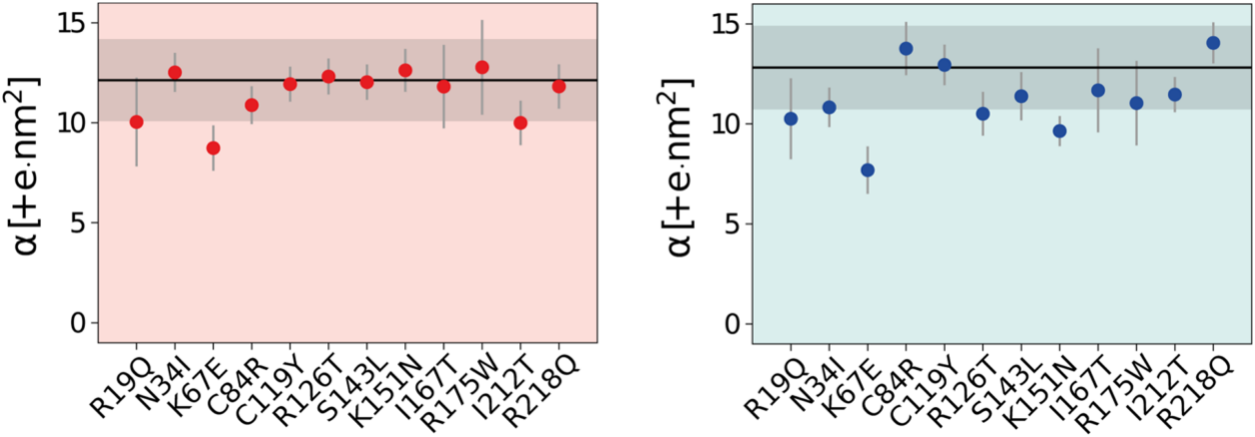
SASA-based binding affinity estimator (α) for SBDS domain I in the open (left) and closed (right) conformation simulations. Black horizontal lines indicate the α value for the WT simulations, while the gray-shaded area represents their standard deviation.

The K67E mutation displays a significant decrease in the α value in both open and closed simulations. This result agrees with the existing literature, as the cryo-EM structure of the 60S-SBDS-eIF6 complex indicates that the K67 residue participates in an electrostatic interaction with the sugar–phosphate backbone of the P-loop of the 60S subunit.

Notably, the α value is affected only by certain mutations. This suggests that their effect may not stem from disrupting the SBSD-60S subunit interaction but rather from impairing another step in the 60S maturation process. For instance, some mutations may alter SBDS flexibility, which is essential in the structural rearrangement required for forming complex 3 in Figure 1A.

### 3.3 Small-Angle X-ray Scattering (SAXS) and Flexibility Analysis of SBDS WT and Mutants

Based on the MD simulation results, we experimentally investigated the impact of mutations on the conformational and structural flexibility of human SBDS using SAXS (Figures 6–8, Supporting Tables 1 and Table 2). We selected one mutation for each domain among the 12 mutations investigated by MD: R19Q (domain I), I167T (domain II), and R175W (domain III). Figure 6 shows the experimental scattering curves, the pair-distance distribution function [P­(r)], and the Kratky plot for all constructs.

**Figure 6:**
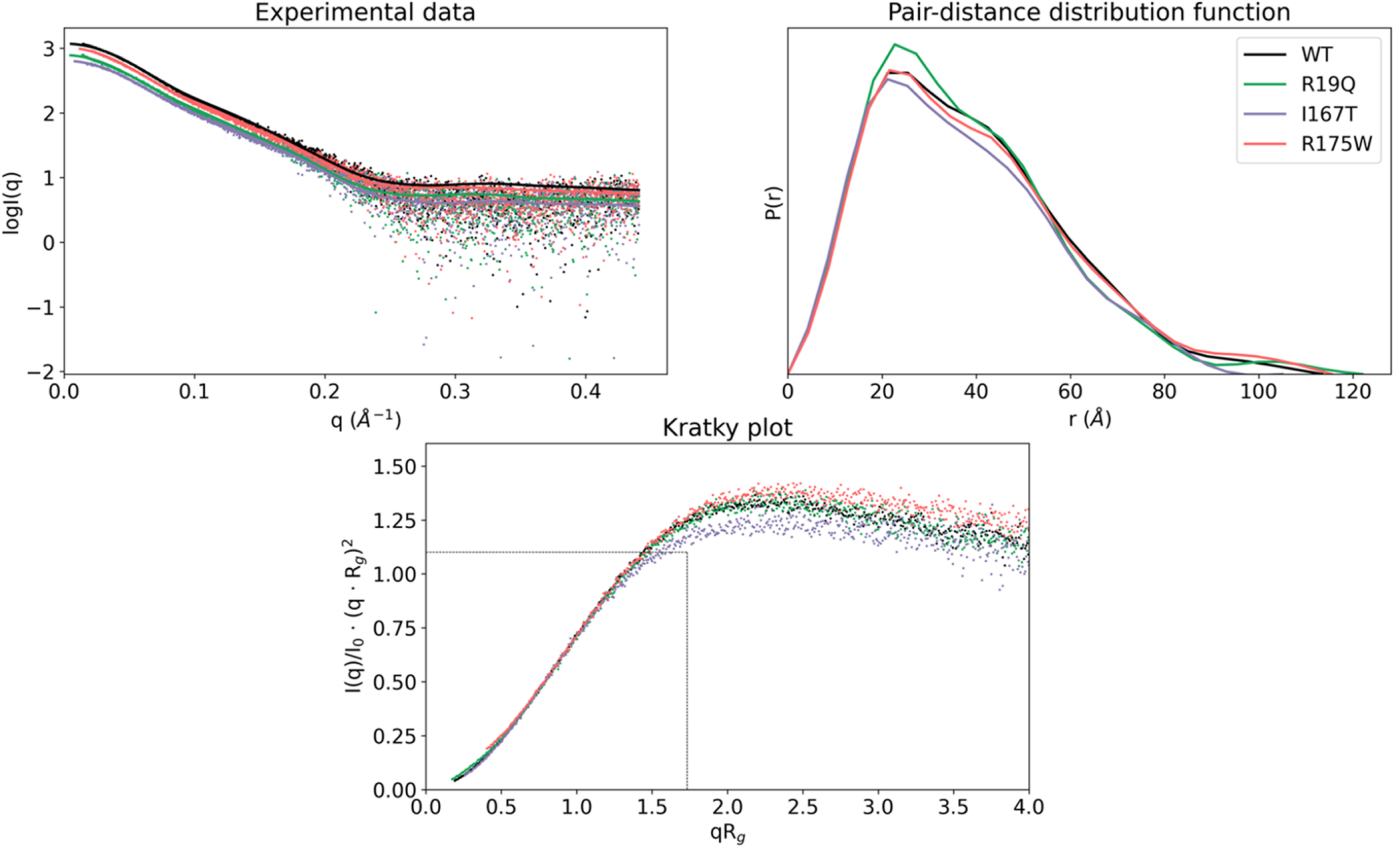
Small-angle X-ray scattering (SAXS) analysis for human SBDS and mutants. Top left panel: Experimental scattering profiles with fitted curves were calculated from MultiFoxs models (solid traces). Top right panel: Pair-distance distribution function plot, P­(r). Bottom panel: Dimensionless Kratky plot, with the intersection of the dotted black trace representing the reference value for bovine serum albumin (BSA). Experimental data for WT, R19W, I167T, and R175W are colored in black, green, violet, and pink, respectively.

**Table 2:** Ensemble Organization of the Top 100 Best-Scoring State *n* Models Based on the SAXS Data for SBDS WT and Mutants R19Q, I167T, and R175W

state	conformation	fraction	*R*_g_ (Å)	*D*_max_ (Å)	angle between domains (°)	χ^2^
WT						
0	0	1	28.4	110.1	122	6.6
3	1	0.08	24.4	76.6	100	4.3
	2	0.77	28.1	93.1	125	
	3	0.15	31.2	107.8	159	
R19Q						
0	0	1	28.4	110.1	122	6.3
3	1	0.17	23.8	88.4	99	5.5
	2	0.22	28.9	95.4	118	
	3	0.61	31.3	108.0	160	
I167T						
0	0	1	28.4	110.1	122	2.0
3	1	0.19	23.9	73.8	97	1.1
	2	0.47	28.3	93.8	121	
	3	0.34	31.4	106.8	162	
R175W						
0	0	1	28.4	110.1	122	7.2
3	1	0.12	24.7	92.5	102	6.0
	2	0.44	28.8	96.6	122	
	3	0.44	30.8	106.3	157	

The maximum value of 1.1 at √3 for *q* × *R*_g_ in the Kratky plot indicates a globular and compact protein, like the bovine serum albumin (BSA) protein used as the standard in the SAXS experiment. All curves displayed a hyperbolic plateau, suggesting a highly flexible protein, consistent with previous descriptions for the *Archaeoglobus fulgidus* ^50^ and *yeast* SBDS^25^ orthologues. Additionally, the P­(r) plots for all of the constructs exhibited a distinct shoulder in the range of 40–50 Å, more pronounced in the mutant proteins than in the WT. This feature suggests an elongated protein structure with distinct domains connected by flexible linkers, indicative of potential conformational variability.

To further investigate the conformational heterogeneity of SBDS WT and its mutants in solution, we used the MultiFOXS program,^45^ as detailed in Section 2. The results are summarized in Figures 7 and 8 and Table 2.

**Figure 7:**
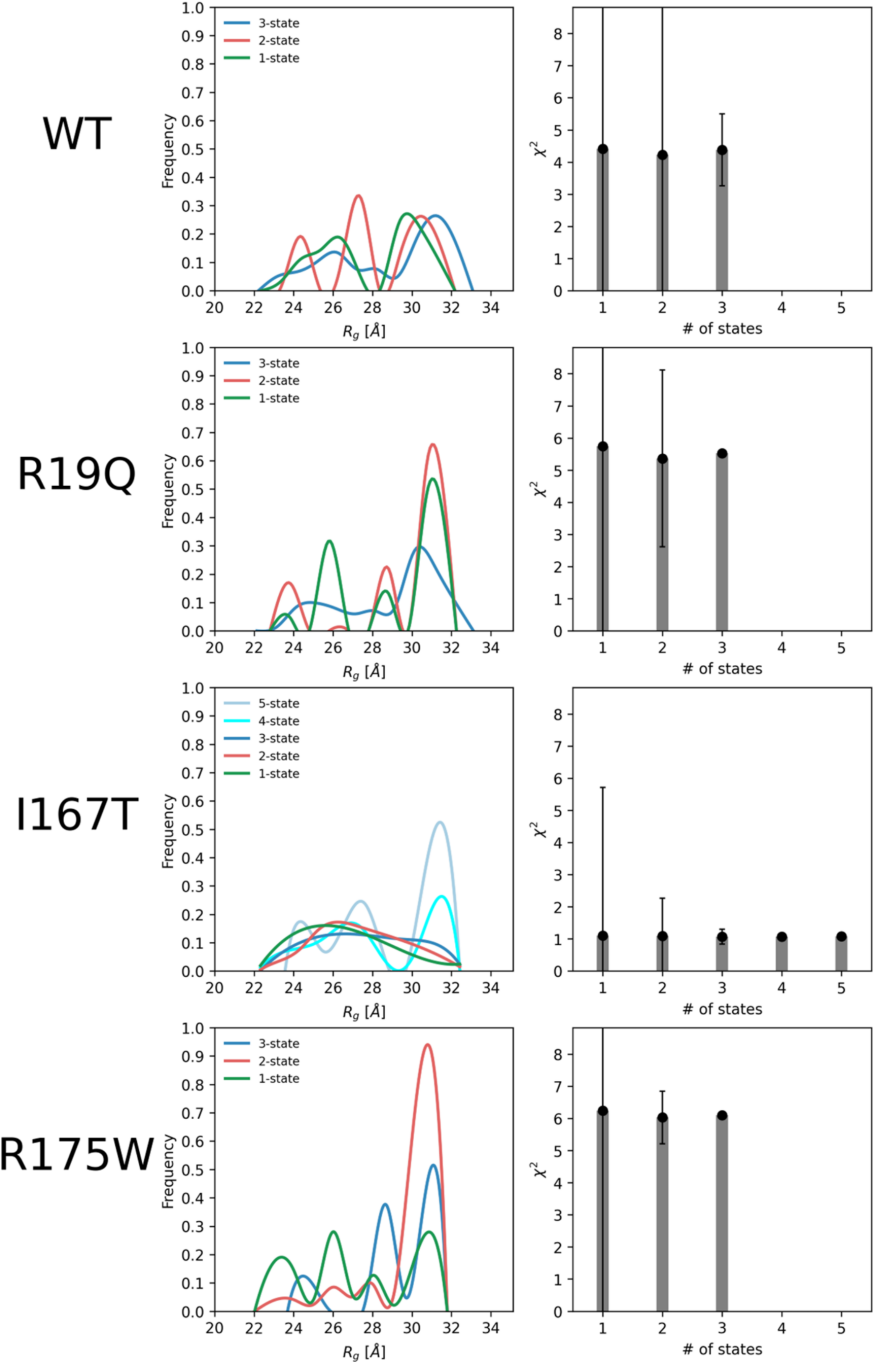
Flexibility analysis of the SBDS ensemble and its mutants. Distribution of the radius of gyration (*R*_g_) in the initial pool of random structures overlapped with those of the final conformational subensembles (left panel). Agreement with experimental data expressed as χ^2^ (and its standard deviation) for each model across all constructs (right panel).

**Figure 8:**
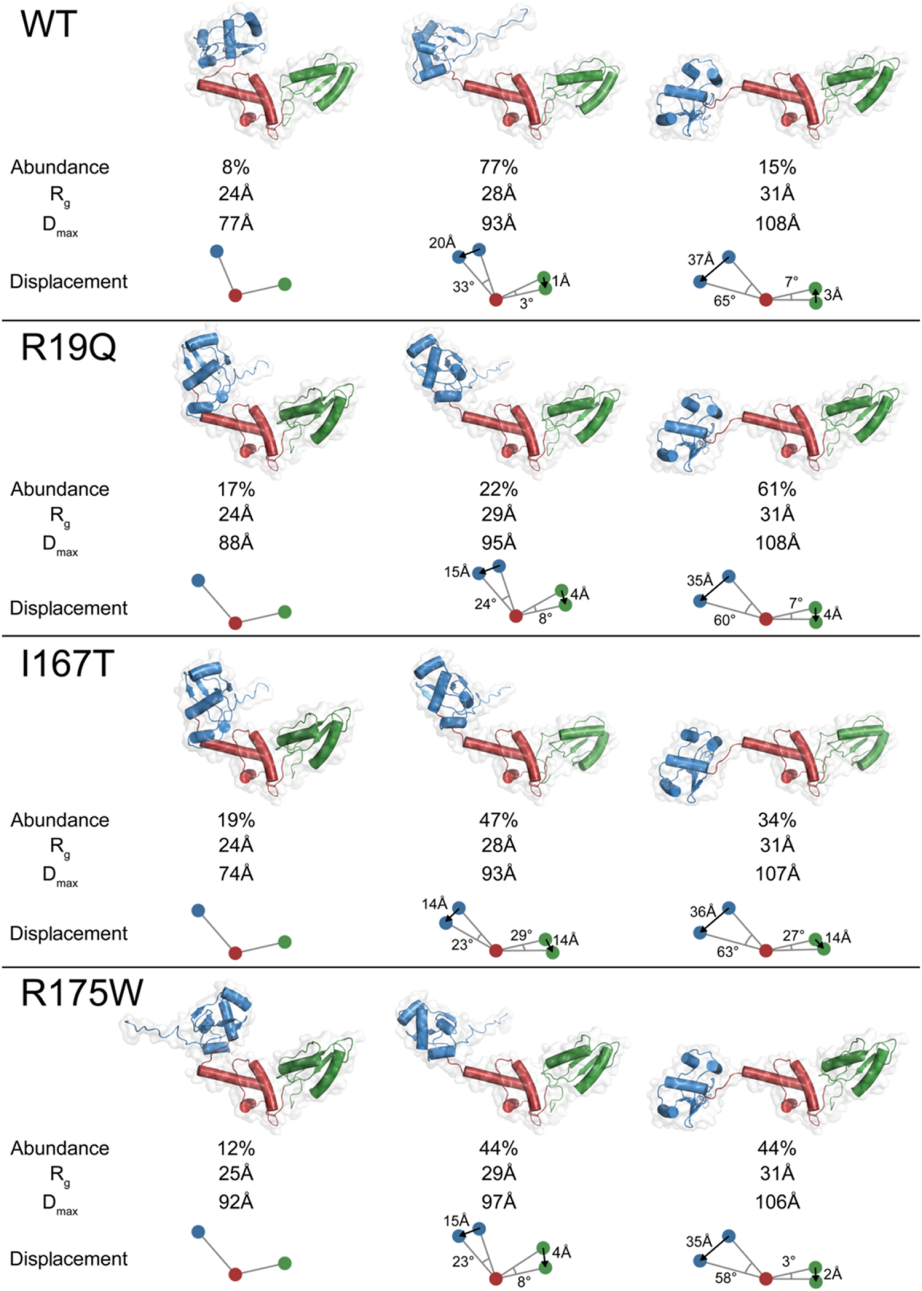
SAXS-based analysis of the SBDS and mutants in solution. The ensemble of conformations comprises a compact (left), stretched (middle), and relaxed (right) states. The percentage abundances, *R*_g_, and maximum dimension (*D*_max_) for each conformation are indicated. Color scheme: domain I (blue), domain II (red), and domain III (green). The displacement of domains I and III (their center of mass), measured in terms of distance and angle relative to the central domain II (center of mass), is the reference against the compact state.

Figure 7 shows the *R*_g_ distribution for the top 100 scored state models for all constructs. The state variable *n*, which ranges from 1 to 5, represents an ensemble described by *n* conformations. The corresponding χ^2^ agreement with the experimental data is reported for each analyzed state. All constructs, with the exception of I167T, were best described by a three-state model, consistent with previous findings for *A. fulgidus* ^50^ and *yeast* SBDS^25^ orthologues. The three-state model (an ensemble comprising three conformations) provided a more accurate *R*_g_ distribution and a better fit to the experimental data, as evidenced by the lower χ^2^ value and relatively smaller standard deviation. For the WT and all mutants, these ensembles include a *compact (conformation 1)*, a *stretched (conformation 2)*, and a *relaxed* conformation *(conformation 3)*. Table 2 provides the percentage of each conformation along with its corresponding *R*_g_ and *D*_max_ values.

Table 2 and Figure 8 illustrate the conformational ensemble of the studied SBDS proteins. By analyzing the values presented above, the following observations can be made:

(i) The R19Q mutant shows a significantly higher proportion of relaxed conformation (61%) compared to the WT (15%), indicating that this mutation drives SBDS toward a more relaxed overall structure. With respect to the compact conformation, in the stretched conformation, domain I is displaced downward by 24° and 15 Å (33° and 20 Å for WT), while domain III shifts in the same direction by 8° and 4 Å (3° and 1 Å in the same direction for WT). In the relaxed conformation, domain I undergoes a larger rotation of 60° and 35 Å (65° and 37 Å for WT), with domain III moving in the same direction by 7° and 4 Å (7° and 3 Å in the opposite direction for WT). In the relaxed conformation, domain I undergoes a larger rotation of 60° and 35 Å (65° and 37 Å for WT), with domain III moving in the same direction by 7° and 34 Å (7° and 3 Å in the opposite direction for WT). These shifts suggest that the R19Q mutation increases the flexibility and movement of domain I more than domain III, similarly to the WT. This result aligns with the MD simulations conducted on the same mutant. As shown in the free-energy surface in Figure 4A, the R19Q mutant can have a larger *R*_g_ compared to the WT, as well as a larger angle between the domains.
(ii) The I167T mutant exhibits a higher proportion of compact conformation (19%) compared to the WT (9%), similar to the R19Q mutant. However, in contrast to R19Q, the 167T mutant transitions more to a stretched than a relaxed conformation, once again consistent with the performed MD simulations. Based on the computed FES, this mutant shows a smaller *R*_g_ relative to the WT and, at least in the open run simulation, a higher interdomain angle. Concerning the displacements relative to the compact conformation, in the stretched conformation, domain I is displaced downward by 23° and 14 Å, while domain III shifts significantly in the same direction by 29° and 14 Å. In the relaxed conformation, domain I undergoes a large rotation (63° and 36 Å), with domain III also moving substantially (27° and 14 Å). Hence, as predicted from the MD simulations (Table 1), the I167T mutation increases flexibility in both domain I–II and the linker II–III, making the protein more dynamic. This is further evidenced by the different proportions of stretched (47%) and relaxed (34%) conformations compared to the WT (77 and 15%, respectively), indicating a potential disruption in the ribosome maturation process due to altered domain movements.
(iii) The R175W mutant shows a distribution of conformations that includes both stretched (44%) and relaxed (44%) states, which is significantly different from the WT, which exists predominantly in the stretched state (77%). Similarly to the observation from the MD simulations, where R175 presents a significantly larger interdomain angle compared to the WT, the MultiFoxs analysis suggests that the mutation increases the proportion of the open conformation. In the stretched conformation, relative to the compact state, domain I is displaced downward by 23° and 15 Å, while domain III shifts slightly less by 8° and 4 Å. In the relaxed conformation, domain I experiences a large rotation (58° and 35 Å), with a smaller movement of domain III (3° and 2 Å). The R175W mutation also causes larger movements in domain I compared to domain III, like the WT and R19Q, but with increased flexibility and displacement.

Across all mutants, SAXS data indicate that domain I exhibits larger movements compared to domain III, suggesting that the flexibility in the linker between domains I and II is consistently higher than that between domains II and III. According to the RMSF plots, mutants showed a similar effect on linker flexibility during the simulations (Figure 3). The increased flexibility observed in the mutants, especially in domain I, indicates that these mutations may impact the structural rearrangements required for SBDS function in the 60S subunit. Notably, the I167T mutation has a significant impact on both domains I and III, suggesting a more pronounced disruption of SBDS structure and potentially its interaction with EFL1 as previously evidenced in Gijsbers et al.^25^ Collectively, these observations highlight how specific mutations in SBDS alter its conformational landscape, potentially affecting its role in ribosome biogenesis and contributing to the pathogenesis of SDS.

## 4 Conclusions

By integrating comparative MD simulations with SAXS-based investigation on SBDS WT and various mutants, this study provides insights into the molecular mechanisms underpinning SDS pathogenesis, summarized in Figure 9 and Table 3. The results indicate that different mutations may affect (i) the interaction of SBDS with the 60S subunit, (ii) the binding of SBDS for EFL1, and (iii) the structural rearrangements of SBDS associated with EFL1 binding. Methodologically, we introduced a new parameter, named α, which serves as an indicator of how specific mutations influence SBDS interaction with the 60S subunit and can be useful for investigating similar systems. The findings from previously published affinity data, combined with the SAXS experimental results presented here, validate the observations from the MD simulations. We propose a structural rationale for the weakened interaction of the I167T, R175W, and I212T mutants with EFL1, as reported by Gijsbers et al.^25^ Additionally, our SAXS data reveal that R19Q, I167T, and R175W mutants exhibit altered relative abundances of distinct conformational states of SBDS in solution, corroborating the computational analyses. Overall, this combined computational and experimental approach provides a comprehensive understanding of how specific SBDS mutations influence its structural dynamics and binding properties. These findings provide valuable insights into the functional role of SBDS and its involvement in ribosome biogenesis.

**Figure 9:**
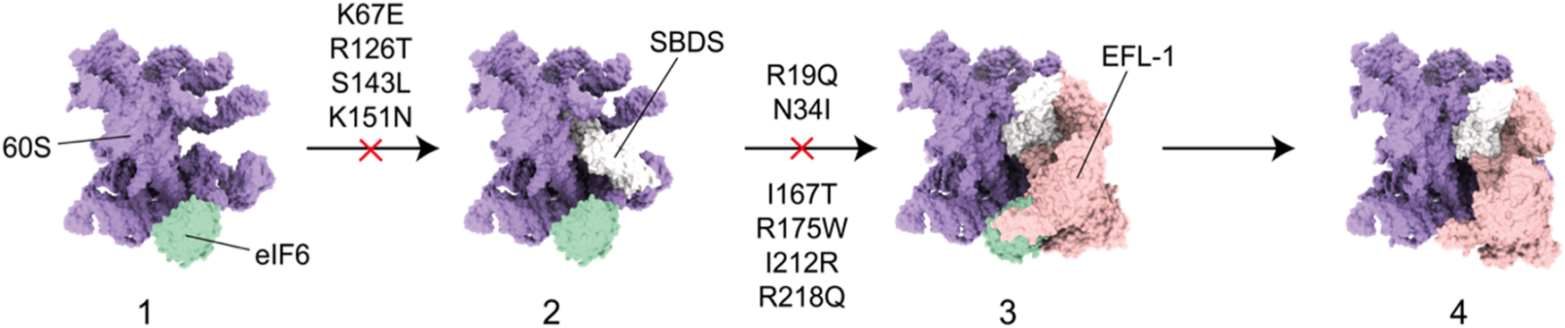
Overview of the effect of the investigated mutations on the ribosome maturation steps emerging from the combined MD/SAXS investigation presented in this paper.

**Table 3:** Main Findings Obtained in This Study Were for Each Investigated SBDS Mutant

Mutation	Hypothesis from the literature	References	Insights from this study	Hypothesis from this study
R19Q	Affects the electrostatic potential of SBDS and its interaction with EFL1	Shammas et al.^48^; Gijsbers et al.^25^	Decrease in the α value	Mutation likely affects SBDS interdomain dynamics weakening the interaction with EFL1
			Observed protein opening in FES favoring the relaxed conformation	
N34I	Affects the electrostatic potential of SBDS	Nicolis et al.^6^; Costa and Santos^7^	Increase in the α value (favors the open state)	Mutation likely affects SBDS interdomain dynamics and/or interaction with the 60S subunit
K67E	Affects interaction with the 60S P-loop	Boocock et al.^3^; Weis et al.^20^	Significant decrease in α values for both open and closed runs	Mutation likely affects SBDS interaction with the 60S subunit or EFL1
C84R	Disrupt SBDS folding	Kuijpers, 2005 ^51^; Finch et al.^26^	No significant insight from this study	No conclusion can be drawn from this study
C119Y	Unknown	Finch et al.^26^	No significant insight from this study	No conclusion can be drawn from this study
R126T	Affects the electrostatic potential of SBDS and its interaction with EFL1	Boocock et al.^3^; Gijsbers et al.^25^	No significant insight from this study	No conclusion can be drawn from this study
S143L	Affects the electrostatic potential of SBDS and its interaction with EFL1	Shammas et al.^48^; Gijsbers et al.^25^	No significant insight from this study	No conclusion can be drawn from this study
K151N	Stabilization of the open conformation of SBDS through interaction with the tip of helix 69	Finch et al.^26^	Domain II is less flexible than in WT (RMSF)	Mutation probably affecting SBDS interdomain dynamics
I167T	Mutation weakening binding to EFL1	Nakashima et al.^5^; Gijsbers et al.^25^	Domain III is less flexible (RMSF open)	Mutation likely affects SBDS interdomain dynamics weakening the interaction with EFL1
			Relaxed conformation is more abundant (SAXS)	
R175W	Mutation disrupting binding to EFL1	Erdos 2006 ^52^; Gijsbers et al.^25^	Domain III is slightly less flexible (RMSF open)	Mutation likely affects SBDS interdomain dynamics weakening the interaction with EFL1
			Relaxed conformation more abundant (SAXS)	
I212T	Mutation disrupting binding to EFL1	Boocock et al.^3^; Gijsbers et al.^25^	Domain III is slightly less flexible (RMSF open and closed)	Mutation likely affects SBDS interdomain dynamics weakening the interaction with EFL1
R218Q	Mutation stabilizing the open conformation of SBDS through interaction with the tip of helix 69	Finch et al.^26^; Weis et al.^20^	Domain III is less flexible (RMSF open)	Mutation probably affecting SBDS interaction with the ribosome

## Supplementary Material



## Data Availability

The MD raw data produced in this work (trajectories and clustering) are freely available on a Zenodo repository at the web address 10.5281/zenodo.13981290. The SAXS data for WT, R19Q, I167T, and R175W have been deposited and validated in the Small Angle Scattering Biological Data Bank (https://www.sasbdb.org) under the codes SASDVC4, SASDVD4, SASDVE4, and SASDVF4, respectively.

## Notes

Time dependence of the RMSD values computed on the Cα atoms for selected mutants (Figure S1); cosine content values of the first three PCs for the open and closed R175W trajectories (Figure S2); cosine content for three PCs from additional replicas of R19Q, I167T, R175W, and WT (Figure S3); time-dependent RMSD of α-carbons for SBDS domains (Figure S4); dendrograms of Jensen–Shannon divergence for open and closed trajectories (Figure S5); difference in SASA per charged residue between closed mutant runs and closed WT (Figure S6); open and closed conformation of PDB 2KDO used as MD starting points (Figure S8); SAXS data collection and structural parameters (Table S1) (PDF)
