# Supplementary material for: Structural Implications of Missense Point Mutations in Shwachman–Bodian–Diamond Syndrome Protein (SBDS): A Combined SAXS/MD Investigation
Source: ACS Omega. 2025 Aug 1;10(31):35103–18. doi: 10.1021/acsomega.5c04764 (PMC12355251; doi:10.1021/acsomega.5c04764)
Supplement: Supplementary file 1 [file ao5c04764_si_001.pdf]

Supporting Information

Structural Implications of Missense Point Mutations in Shwachman-Bodian-Diamond syndrome protein (SBDS): a combined SAXS/MD investigation

Giovanni Mattiotti[1,2 Ω], Vittoria Nanna[3Ω], Marco Giulini[1,2], Domenico Alberga[3], Giuseppe Felice Mangiatordi[3], Nuria Sánchez-Puig[4], Michele Saviano[5], Luca Tubiana[1,2], Raffaello Potestio[1,2], Gianluca Lattanzi*[1,2], and Dritan Siliqi*[3]

[1]Physics Department, University of Trento, via Sommarive 14, I-38123 Trento, Italy

[2]INFN-TIFPA, Trento Institute for Fundamental Physics and Applications, I-38123 Trento, Italy

[3]CNR - Istituto di Cristallografia, Via Amendola 122/o, I-70126 Bari

[4]Instituto de Química, Universidad Nacional Autónoma de México, Circuito Exterior s/n, Ciudad Universitaria, México City 04510, México

[5]CNR - Istituto di Cristallografia, URT Caserta, Via Vivaldi 43, I-81100 Caserta, Italy

[Ω] G.M. and V.N. contributed equally to this study.

*Corresponding Author: gianluca.lattanzi@unitn.it and dritan.siliqi@cnr.it

Supplementary Figures

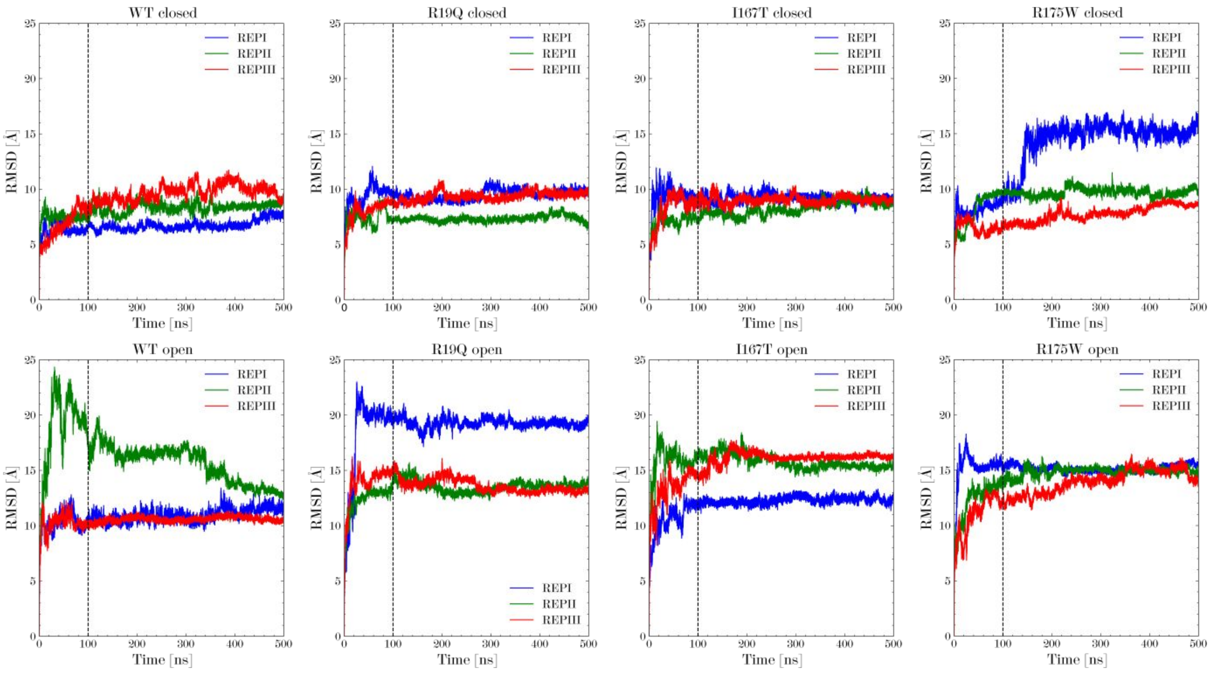

Figure S1. Time-dependence of the RMSD values computed on the Cα atoms for selected SBDS mutants - WT, R19Q, I167T, and R175W - for which three independent replicas were performed. The black dashed line separated the equilibration phase from the sampling one.

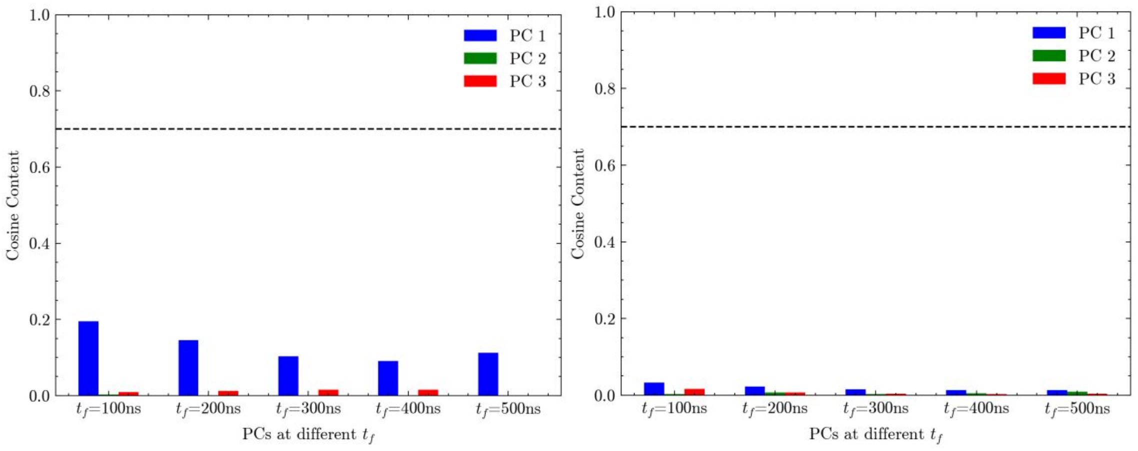

Figure S2. Cosine content values of the first 3 PCs, for the open (left) and closed (right) trajectory of R175W-mutated SBSB. The dashed back line highlights the value 0.7, indicated by Hess [10.1103/PhysRevE.65.031910] as the threshold below which the cosine content must be considered dangerously high. For values higher than 0.7, the simulation collective motions are similar to random diffusion. Each value corresponds to a different cutoff applied to the length of the trajectory: the higher it is, the longer is the considered block for the cosine content calculation. A decreasing trend of this value, as observed for PC1, is a sign of an equilibrated trajectory."

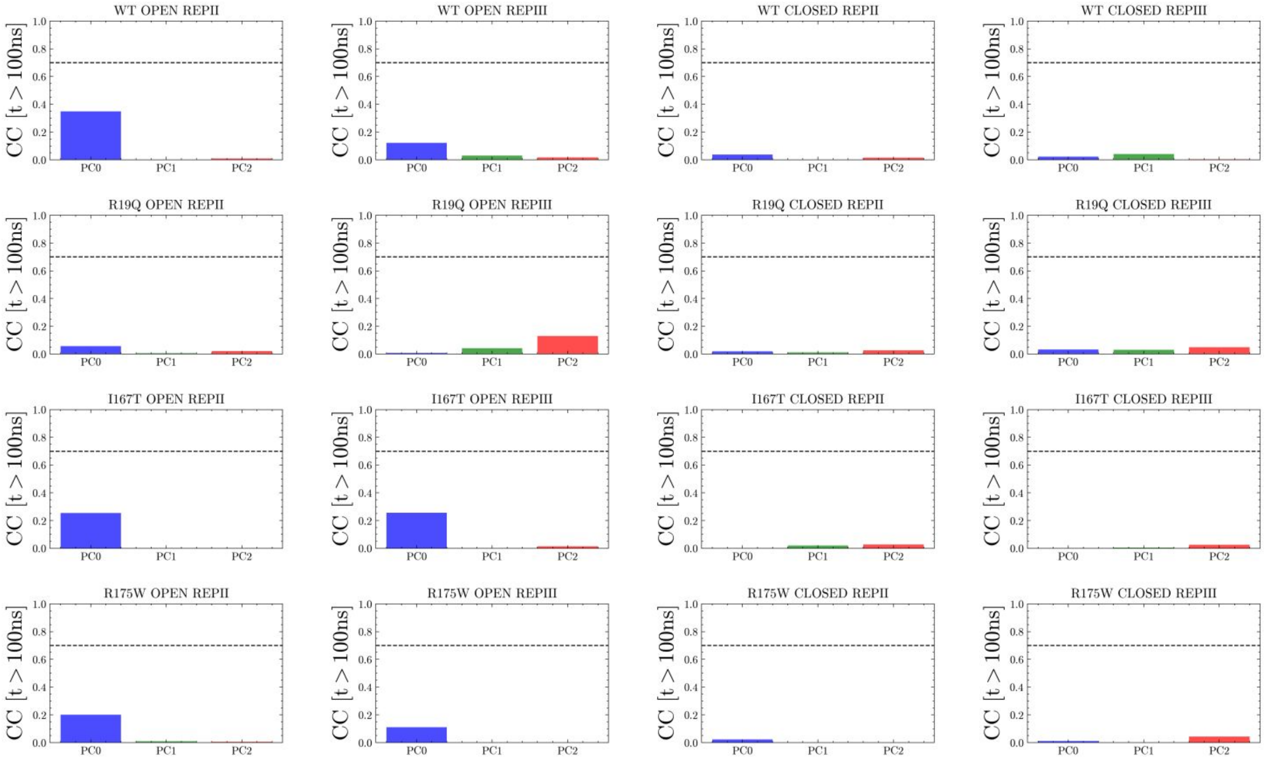

Figure S3. Complementary calculations of the cosine content for the 3 main PCs, relative to the 2 additional replicas simulated for the mutants R19Q, I167T, R175W and theWT. The values are calculated considering the full 500ns-long trajectories. The trend clearly shows the absence of dangerous values, all of them being below 0.7.

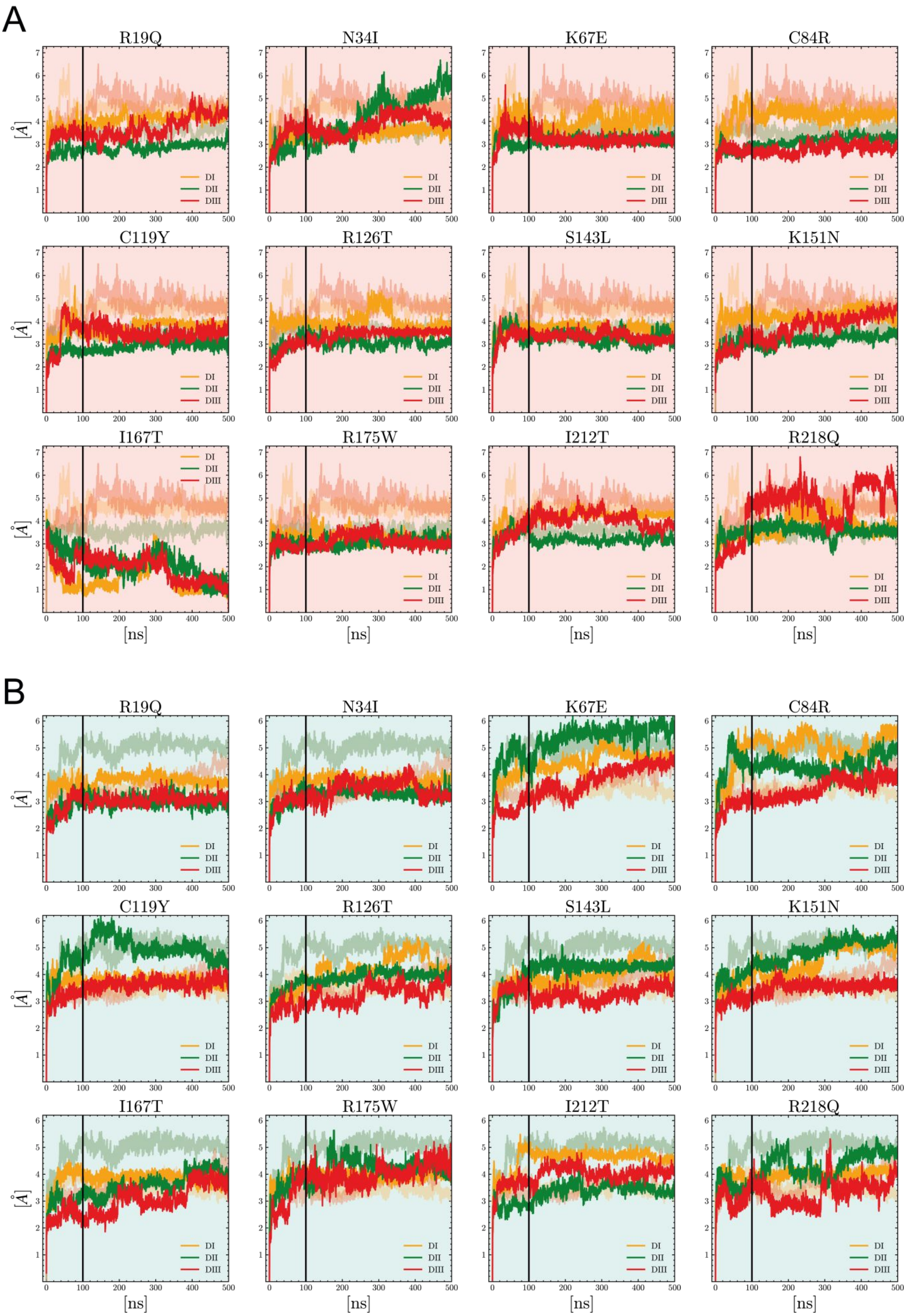

Figure S4. Time-dependence of the RMSD values returned by the open (A) and closed (B) trajectories computed on the α carbons for each domain (DI, domain I, in yellow; DII,

domain II, in green; DIII, domain III, in red) separately. In each plot, the WT curves are plotted in the background with the domains coloured according to the same scheme but in a lighter shade.

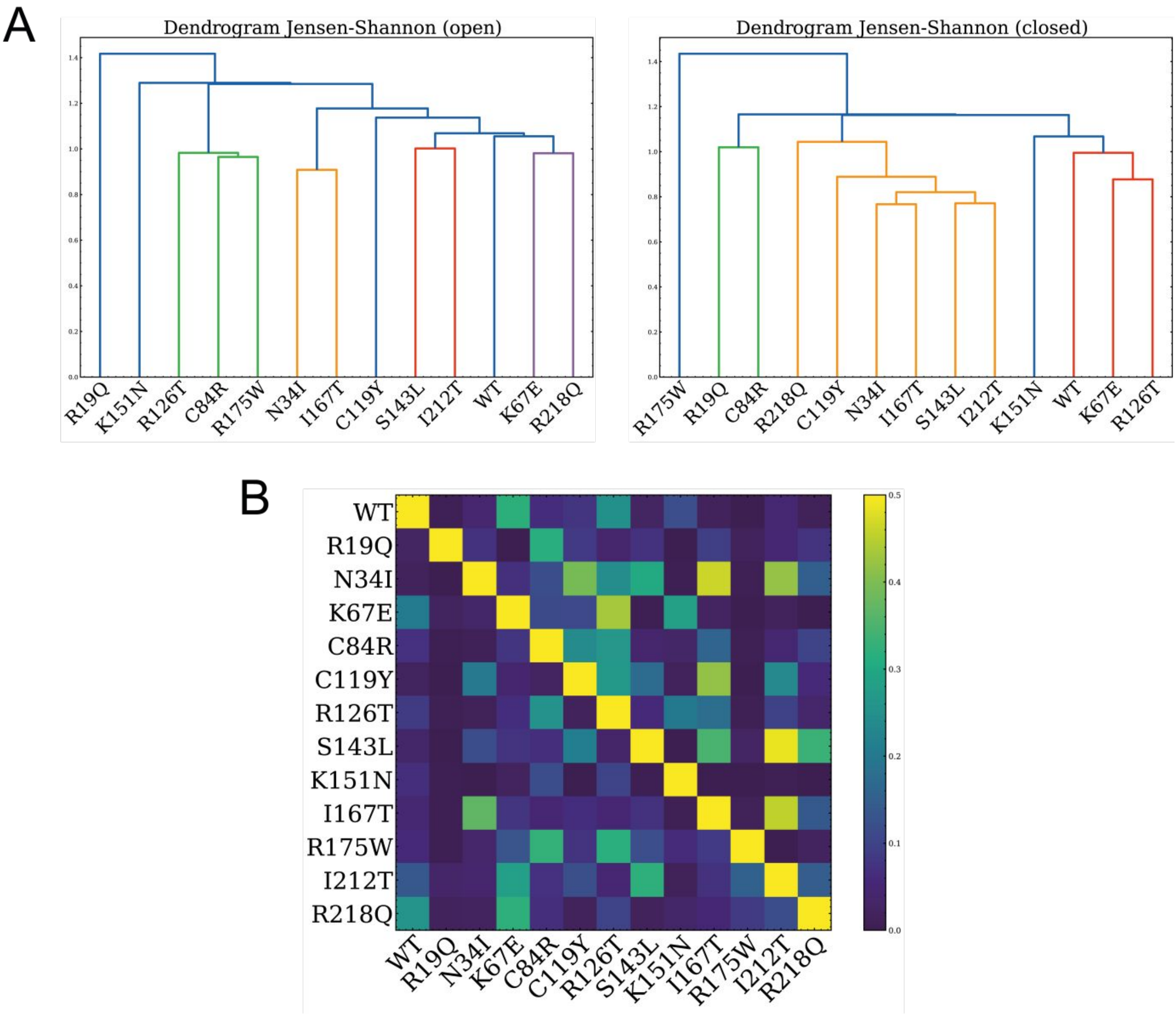

Figure S5. (A) Dendrograms presenting the Jensen-Shannon divergence of SBDS variants calculated on the trajectories resulting from the open (left) and closed (right) states. (B) Jensen-Shannon divergence-based similarity matrix of the free-energy landscape sample in the open (lower-left values) and closed (upper-right values) runs. The colour map was compressed in the range [0, 0.5] to highlight the relative differences, although in principle the divergence can take values in [0, 1].

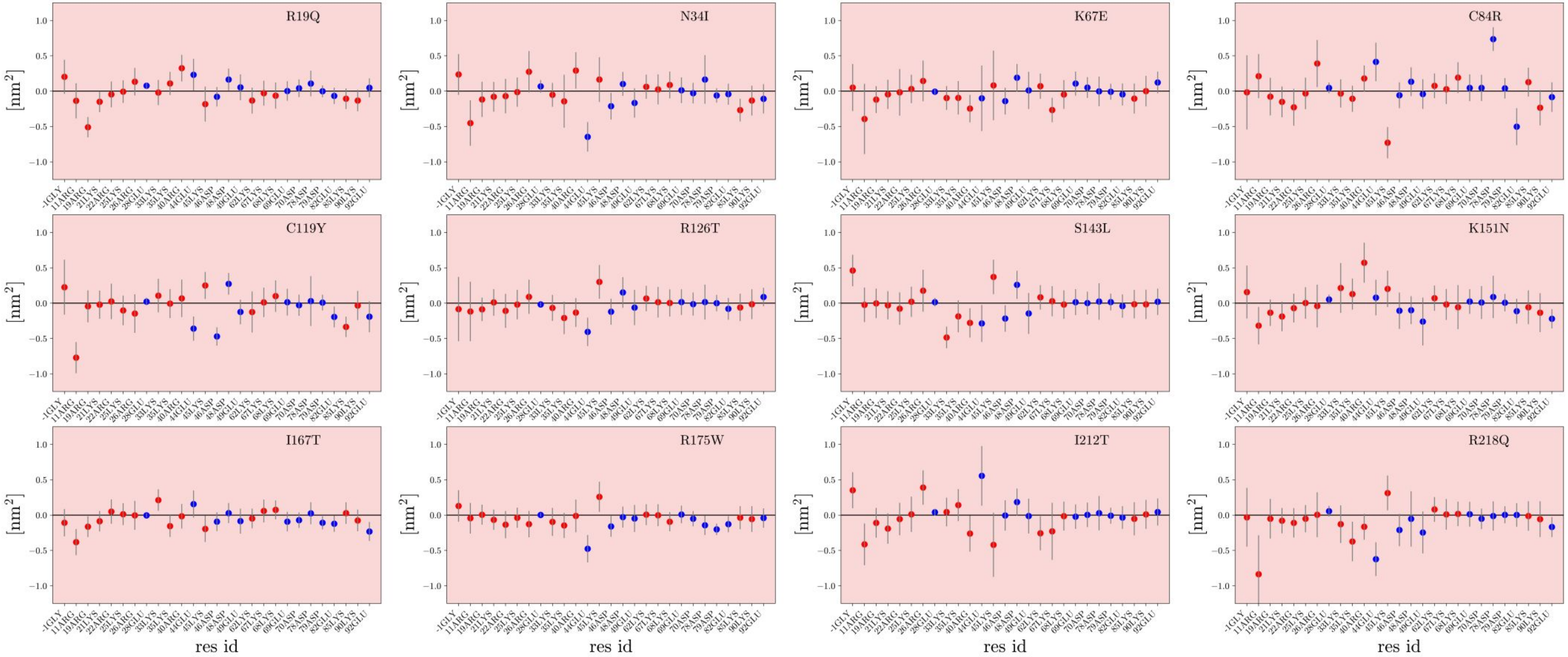

Figure S6. Difference computed between the SASA of each charged residue in the open mutant runs and the open WT run. The positively and negatively charged residues are represented by red and blue dots, respectively.

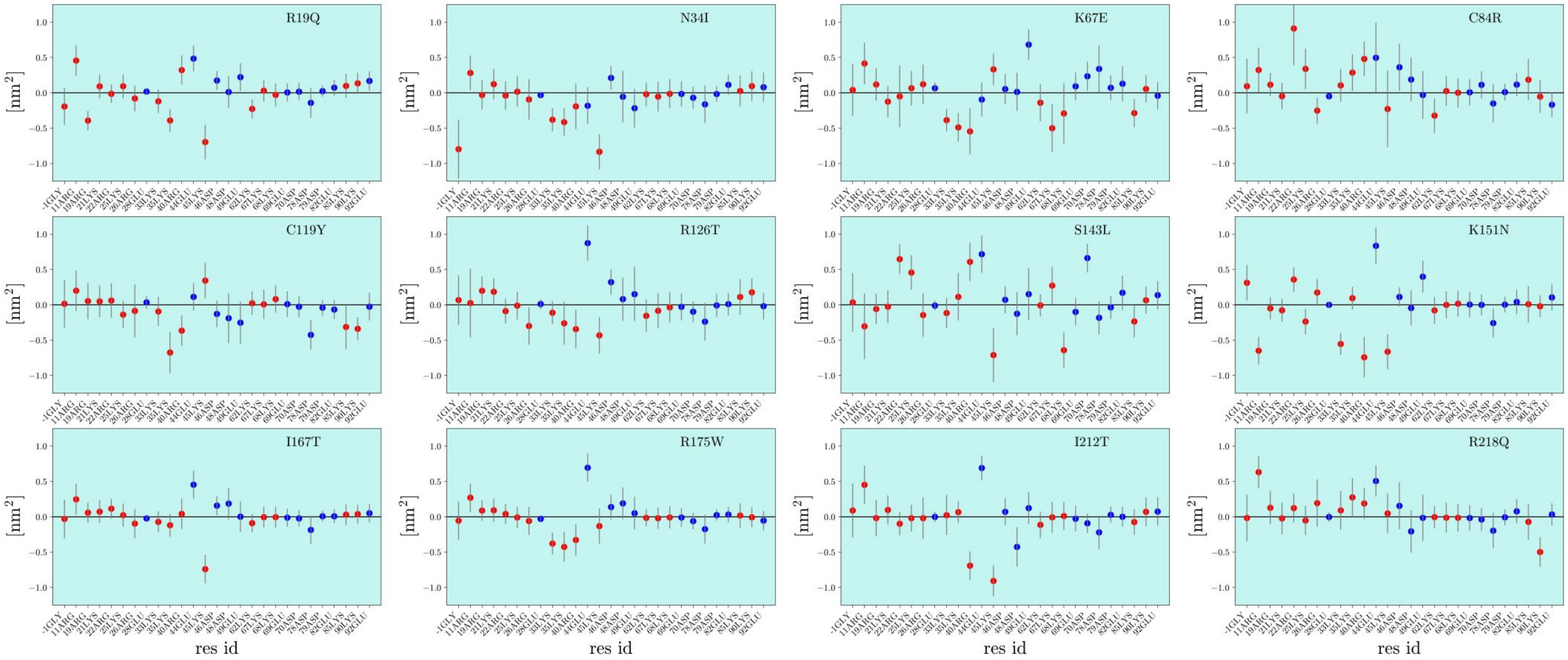

Figure S7. Difference computed between the SASA of each charged residue in the closed mutant runs and the closed WT run. The positively and negatively charged residues are represented by red and blue dots, respectively.

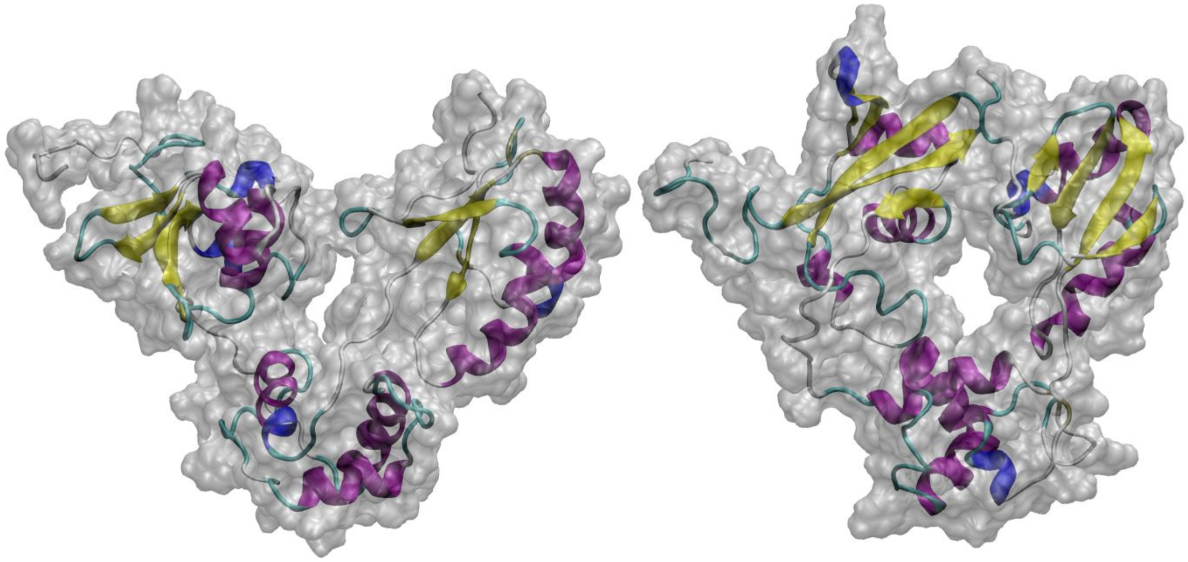

Figure S8. Open (left) and closed (right) conformations, corresponding to conformer II and V of PDB 2KDO, used as starting point for the MD simulations presented in this work. The secondar structure motifs are colored differently, while a transparent representation of the molecular surface, built from the Van der Waals radii of the atoms, is shown in grey.

Supplementary Tables

Table S1: SAXS data collection and structural parameters

**Data collection parameters**				
Beamline	P12, DESY			
Detector	Pilatus 6M			
Beam size (mm)	0.2 x 0.05			
Energy (keV)	8.0			
Sample-to-detector distance (m)	3.0			
s range (A^{-1})	0.0025 - 0.6			
Exposure time (s)	0.5 (1800 frames)			
Temperature (K)	293			
Data collection mode	SEC online (50 ul superdex 75 increase 5 150gl flow 0.3 P 22.5)			
**Structural parameters**	WT	R19Q	I167T	R175W
Concentration range (mg ml^{-1})	11.0	13.8	13.8	13.5
s Interval for Fourier inversion ($Å^{-1}$)	0.009 - 0.247	0.015 - 0.231	0.009 - 0.247	0.017 - 0.242
R_g [from P(r)] (Å)	30.39 ± 0.04	29.85 ± 0.06	28.81 ± 0.06	31.14 ± 0.10
R_g [from Guinier approximation] (Å)	30.15 ± 0.10	29.17 ± 0.15	28.83 ± 0.11	30.58 ± 0.16
sR_g limits [from Guinier approximation]	0.49 - 1.23	0.59 - 1.29	0.29 - 1.29	0.64 - 1.28
D_{max} (Å)	113.5	122.0	105.0	115.5
Porod coefficient	2.2	2.1	1.9	2.1
Porod volume estimate (nm^3)	50	46	44	48
Estimated Molecular Mass (kDa)	29.4 - 31.3	27.1 – 28.8	25.9 – 27.5	28.2 - 30
Molecular Mass from sequence (kDa)	26.6			
Modelling Ambiguity	2.0 – 2.8 (might be ambiguous)			
**SASBDB**	SASDVC4	SASDVD4	SASDVE4	SASDVF4
**Software employed**				
Primary data reduction	P12 online software tools			
Data processing	ScÅtter IV / ATSAS 3.0.4			
Computation of model intensities	CRYSOL (ATSAS)			
Modelling	MultiFoxs			
